# Origins of spontaneous activity in the degenerating retina

**DOI:** 10.3389/fncel.2015.00277

**Published:** 2015-07-29

**Authors:** Stuart Trenholm, Gautam B. Awatramani

**Affiliations:** ^1^Friedrich Miescher Institute for Biomedical ResearchBasel, Switzerland; ^2^Department of Biology, University of VictoriaVictoria, BC, Canada

**Keywords:** retinal degeneration, oscillations, AII amacrine cells, gap junctions, Na^+^ channels, retina, bipolar cells, ganglion cells

## Abstract

Sensory deafferentation resulting from the loss of photoreceptors during retinal degeneration (rd) is often accompanied by a paradoxical increase in spontaneous activity throughout the visual system. Oscillatory discharges are apparent in retinal ganglion cells in several rodent models of rd, indicating that spontaneous activity can originate in the retina. Understanding the biophysical mechanisms underlying spontaneous retinal activity is interesting for two main reasons. First, it could lead to strategies that reduce spontaneous retinal activity, which could improve the performance of vision restoration strategies that aim to stimulate remnant retinal circuits in blind patients. Second, studying emergent network activity could offer general insights into how sensory systems remodel upon deafferentation. Here we provide an overview of the work describing spontaneous activity in the degenerating retina, and outline the current state of knowledge regarding the cellular and biophysical properties underlying spontaneous neural activity.

## The Degenerating Retina is Intrinsically Noisy

For centuries it has been known that when people suffer vision loss they often experience differing degrees of visual hallucinations (Ffytche, 2009; Schadlu et al., 2009). Some of these hallucinations, described as scintillations or phosphenes, which appear as spontaneous flashes or flickering of light, may originate in the retina (Lepore, 1990; Murtha and Stasheff, 2003). The first physiological evidence that spontaneous activity during retinal degeneration (rd) arises in the retina was found in recordings from neurons in the visual system of a mouse model for rd (Dräger and Hubel, 1978). In this study, it was found that rd was associated with rhythmic activity in neurons in visual cortex and superior colliculus. Upon asphyxiating the eye, it was found that the spontaneous activity disappeared, thus suggesting that the spurious activity was arising in the retina (Dräger and Hubel, 1978). Similar findings of increased spontaneous activity were subsequently reported in superior colliculus recordings from rd rats (Sauvé et al., 2001) and from a different rd mouse model (Ivanova et al., 2015). Physiological recordings in the rd retina have directly revealed an increase in spontaneous spike activity in ganglion cells, despite a reduction or absence of sensory input (Pu et al., 2006; Stasheff, 2008).

The finding of increased spontaneous activity in ganglion cells in retinae with degenerating photoreceptors has now been repeated by many labs and in several different animal strains including rd1 mice (rd caused by a mutation in the Pde6b gene; Margolis et al., 2008; Stasheff, 2008; Borowska et al., 2011; Menzler and Zeck, 2011), rd10 mice (rd caused by a mutation in the Pde6b gene; Goo et al., 2011; Stasheff et al., 2011; Toychiev et al., 2013; Biswas et al., 2014), P23H-1 rats (rd caused by a mutation in the rhodopsin gene; Sekirnjak et al., 2011), and Royal College of Surgeons (RCS) rats (rd caused by a mutation in the Mertk gene; Pu et al., 2006). While increased spiking is observed in ganglion cells in all models, the details regarding the precise timing of photoreceptor degeneration and whether ON or OFF ganglion cell circuits are more affected appears to vary between lines. Here, we focus on experiments performed primarily in fast onset retinal degeneration (rd1) and slow onset retinal degeneration (rd10) mice, since the majority of the work examining the biophysical underpinnings of this spontaneous retinal activity has been undertaken using these lines.

One of the most noticeable features of the spontaneous activity in the rd1 and rd10 retina is that there is a tendency for ganglion cells to fire rhythmically, at a frequency of ~10 Hz (Margolis et al., 2008; Stasheff, 2008; Borowska et al., 2011; Menzler and Zeck, 2011; Stasheff et al., 2011; Yee et al., 2012; Biswas et al., 2014). Such spontaneous activity could impair strategies that attempt to restore sight to blind retinae. Indeed it has been shown that this neural noise impairs the fidelity of synaptic transmission to ganglion cells when bipolar cells are ectopically electrically stimulated (Yee et al., 2012). In order to better understand the spontaneous activity and apply this knowledge to the design of vision restoration strategies, it is important to address two central questions: in which cells is this spontaneous activity arising and what are the biophysical properties that underlie it?

## Spontaneous Activity Arises at the Level of the AII Amacrine/ON Cone Bipolar Cell Network

One possibility is that structural changes and rewiring of retinal circuits may lead to spontaneous activity during photoreceptor degeneration. While gross anatomical changes have been noted in the outer retina in several rd models (Strettoi and Pignatelli, 2000; Strettoi et al., 2002; Jones et al., 2003), only minor morphological changes have been shown to occur in ganglion cells during rd (Mazzoni et al., 2008; Damiani et al., 2012; Lin and Peng, 2013; O’Brien et al., 2014). In rd mice, even after most photoreceptors have degenerated, ganglion cells can still be categorized as either ON, OFF or ON-OFF, based on their stereotypical dendritic stratification patterns in the inner-plexiform layer (Margolis et al., 2008; Mazzoni et al., 2008; Borowska et al., 2011; Damiani et al., 2012; Yee et al., 2012, 2014; Lin and Peng, 2013; O’Brien et al., 2014). These findings, together with the physiological results discussed below, indicate that gross anatomical rewiring of the retinal circuitry is not likely to underlie the emergence of spontaneous activity in the degenerating retina.

Several studies combining electrophysiological and pharmacological approaches suggest that a physiological remodeling of the inner retina appears to give rise to spontaneous activity in ganglion cells. Whole-cell voltage-clamp recordings have revealed that ganglion cells receive spontaneous excitatory and inhibitory oscillatory inputs in rd retinae (Margolis et al., 2008; Borowska et al., 2011; Yee et al., 2012, 2014). When glutamatergic chemical synaptic input to ganglion cells is pharmacologically blocked, spontaneous oscillatory ganglion cell spiking is largely inhibited (Menzler and Zeck, 2011). Therefore, a major source of spontaneous activity in the rd retina must be presynaptic to retinal ganglion cells.

An initial hypothesis was that certain inhibitory amacrine cells, such as wide-field or starburst amacrine cells, which have been shown to exhibit rhythmogenic behavior in wild type retinae (Solessio et al., 2002; Vigh et al., 2003; Petit-Jacques et al., 2005), might drive oscillatory activity in the rd retina (Margolis and Detwiler, 2011; Yee et al., 2012). In this model, activity in amacrine cells would be conveyed to ganglion cells via modulation of glutamate release from bipolar cell axon terminals. Pharmacological assessment of spontaneous activity in retinal ganglion cells, however, did not support this hypothesis. Blocking inhibition was found to increase spontaneous ganglion cell activity, suggesting that spontaneous ganglion cell spiking is primarily driven by excitatory input from presynaptic bipolar cells (Borowska et al., 2011; Menzler and Zeck, 2011). However, this issue was not clear cut as some spontaneous inhibitory inputs to ganglion cells persisted in the presence of glutamate receptor blockers (Borowska et al., 2011; Menzler and Zeck, 2011). While these inhibitory inputs alone were ineffective in driving significant rhythmic output from retinal ganglion cells (Menzler and Zeck, 2011), these findings suggested that an upstream element was active in the absence of glutamatergic signaling.

Which cells presynaptic to ganglion cells are generating the spontaneous activity? Whole-cell recordings were subsequently obtained from bipolar and amacrine cells in the whole mount rd retina. These recordings revealed membrane oscillations, with a frequency of ~10 Hz, in AII amacrine and cone bipolar cells (Borowska et al., 2011; Trenholm et al., 2012) that were similar to those observed in ganglion cells. Applying a cocktail of excitatory and inhibitory chemical synaptic blockers did not inhibit oscillations in AII amacrine or ON cone bipolar cells (Borowska et al., 2011), providing the first evidence of presynaptic intrinsic oscillatory elements. Interestingly, oscillations in AII amacrine cells in rd retinae have also been observed in 200–300 μm thick retinal slices (Choi et al., 2014; Margolis et al., 2014), indicating that a reduced network is sufficient to drive neural oscillations.

In the wild type retina, AII amacrine cells are strongly coupled to the axon terminals of ON cone bipolar cells as well as to other AII amacrine cells (Famiglietti and Kolb, 1975; Strettoi et al., 1992, 1994; Veruki and Hartveit, 2002a, b). In addition, AII amacrine cells also provide glycinergic inhibition to axon terminals of OFF cone bipolar cells and dendrites of OFF ganglion cells (Pourcho and Goebel, 1985; Ivanova et al., 2006; Münch et al., 2009). Thus, if spontaneous ganglion cell activity originates in the AII amacrine/ON cone bipolar cell network, activity would be expected to occur out of phase in neighboring ON and OFF ganglion cells (Kerschensteiner and Wong, 2008). This prediction was recently confirmed by simultaneous measurements of spontaneous activity from neighboring ON and OFF ganglion cells in rd retinae (Margolis et al., 2014; Menzler et al., 2014). Additionally, oscillatory responses of OFF, but not ON, retinal ganglion cells in rd retinae were antagonized when glycinergic synaptic transmission was pharmacologically blocked (Poria and Dhingra, 2015). Thus, there appears to be a general agreement that the AII amacrine/ON cone bipolar cell network is responsible for pacemaker activity in the degenerating retina.

Are other cell types spontaneously active in the degenerating retina? Certain ganglion cells appear to have slightly higher spontaneous firing rates in synaptic blockers in rd retina compared to wild type retina (Sekirnjak et al., 2011), though the mechanism behind this increased activity is unknown. Next, recent calcium imaging studies have found spontaneous rhythmic activity in the outer retina, including in the remaining cell bodies of degenerated photoreceptors and in horizontal cells (Haq et al., 2014). The frequency of the rhythmic spontaneous activity in the outer retina was slower (~3 Hz) than the activity previously described in AII amacrine, cone bipolar, and ganglion cells (~10 Hz). Therefore while the spontaneous activity in the outer retina may modulate activity to some extent in the degenerating retina, the key pacemaker lies at the level of AII amacrine/ON cone bipolar cells. This pacemaker activity can then diversify in different ganglion cell types (Sanes and Masland, 2015) when different presynaptic microcircuits are activated (Yee et al., 2014).

## TTX-Sensitive Na^+^ Channels and Gap Junction Mediated Network Interactions Underlie Pacemaker Activity

**Figure 1:**
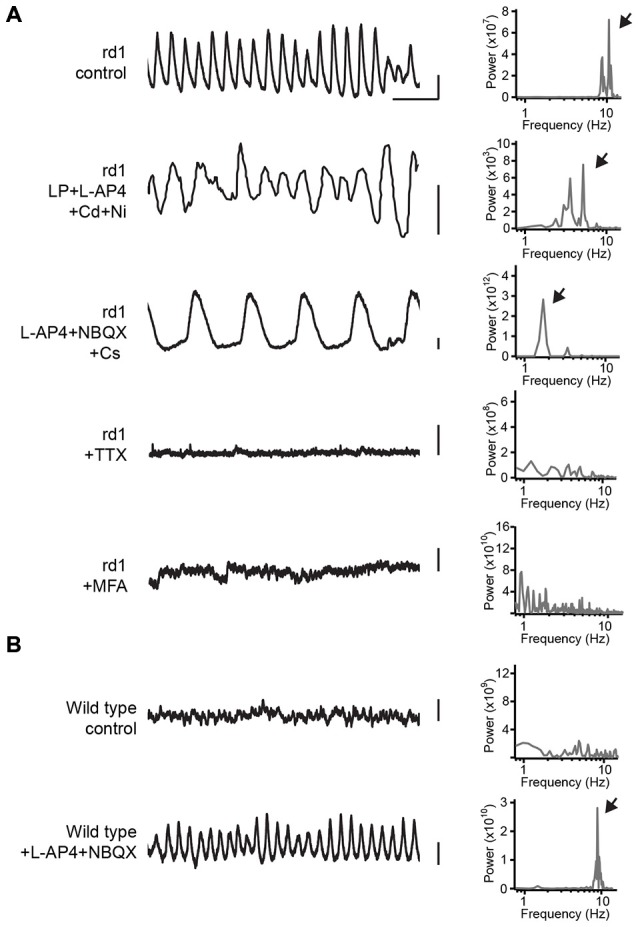
**Pharmacological analysis of spontaneous oscillations in AII amacrine cells. (A)** On the left are shown plots of the membrane potential of AII amacrine cells in rd1 retina in control and in a set of different pharmacological agents. The top trace and bottom three traces were recorded in whole-mount retina. The second trace from the top is adapted from Figure 9C from Choi et al. (2014). A power spectral analysis is shown to the right of each voltage trace, and arrows point to the frequency of the oscillation. **(B)** The same as **(A)**, except for wild type retina, see also data used in Trenholm et al. (2012). Abbreviations: LP, linopirdine dihydrochloride (M-type K^+^ channel blocker); L-AP4, L-(+)-2-amino-4-phosphonobutyric acid (mGluR6 receptor agonist); Cd, cadmium chloride (voltage-gated Ca^2+^ channel blocker); Ni, nickel chloride (voltage-gated Ca^2+^ channel blocker); NBQX, 2,3-dihydroxy-6-nitro-7-sulfamoyl-benzo[f]quinoxaline-2,3-dione (AMPA/Kainate receptor antagonist); Cs, cesium (I_h_ blocker); TTX, Tetrodotoxin (voltage-gated K^+^ channel blocker); MFA, meclofenamic acid (gap junction blocker). Scale bar is 500 ms for *x*-axis; 3 mV for *y*-axis.

To gain an understanding of the biophysical basis for oscillations in the AII amacrine/ON cone bipolar cell network in the rd retina, previous studies have largely relied on pharmacology (see Figure 1 for a summary). Some of the first candidates for driving oscillations were voltage-gated Ca^2+^ channels in bipolar cells, as these have been shown to drive regenerative rhythmic activity in isolated cells (Burrone and Lagnado, 1997; Ma and Pan, 2003; Palmer, 2006). However, since oscillations in AII amacrine cells and ON cone bipolar cells in rd retinae were not blocked upon application of voltage-gated Ca^2+^ channel blockers (Borowska et al., 2011), these bipolar cell conductances cannot be fundamental in driving oscillations in rd retinae. Another possible candidate was the hyperpolarization activated currents (I_h_), which mediate oscillatory activity in other parts of the brain (Gauss and Seifert, 2000). I_h_ is prominent in many cone bipolar cells, but not in AII amacrine cells (Trenholm et al., 2012). Blocking I_h_ leads to membrane hyperpolarization of the AII amacrine/ON cone bipolar cell network (indicating I_h_ is active during ongoing activity), an increase in oscillation amplitude and a decrease in oscillation frequency (Figure 1A; Trenholm et al., 2012). Thus while active conductances in bipolar cell terminals can strongly modulate the amplitude and frequency of spontaneous oscillations in rd retinae, they do not appear to generate oscillations.

In contrast to I_h_ and voltage-gated Ca^2+^ channels, blocking voltage-gated Na^+^ channels with Tetrodotoxin (TTX) completely abolishes oscillatory synaptic input to retinal ganglion cells (Trenholm et al., 2012), as well as completely blocks spontaneous oscillations in both AII amacrine and ON cone bipolar cells recorded in the whole mount retina (Figure 1A; Trenholm et al., 2012) and in slice preparation (Choi et al., 2014; Margolis et al., 2014). Since voltage-gated Na^+^ channels are strongly expressed in AII amacrine cells where they drive spiking responses, but are only weakly expressed by a few bipolar cell types in mouse retina (Pan and Hu, 2000; Cui and Pan, 2008), given the widespread nature of oscillatory inputs to diverse ganglion cell types it is likely that Na^+^ channels in AII amacrine cells are the primary drivers of pacemaker activity.

In addition to voltage-gated Na^+^ channels, gap junctions have also been found to play a critical role in generating oscillations. Consistent with AII amacrine and ON cone bipolar cells being gap junction coupled in rd retinae (similar to what has been shown in wild type retinae, as described above), strongly modulating the membrane potential (from −80 to 0 mV) does not abolish spontaneous membrane oscillations (Borowska et al., 2011) and gap junction blockers significantly increase input resistance. Importantly, it was found that applying gap junction blockers eliminated membrane oscillations for both AII amacrine and ON cone bipolar cells recorded in whole mount retinae (Figure 1A; Trenholm et al., 2012) as well as for AII amacrine cells in retinal slices (Choi et al., 2014). Thus, individually, neither AII amacrine nor ON cone bipolar cells appear responsible for generating the pacemaker activity. Moreover, gap junction blockers also inhibit oscillatory activity in downstream ganglion cells (Menzler and Zeck, 2011; Trenholm et al., 2012), and knocking out connexin 36, which makes up gap junctions in many retinal cell types including AII amacrine cells, greatly reduces spontaneous input to retinal ganglion cell in rd retinae (Ivanova et al., 2015). Therefore, it appears that gap junctions play a critical role in generating oscillatory activity in the degenerating retina.

While the general biophysical framework of network oscillations has been well established, the precise mechanism whereby gap junctions enable oscillations to arise is not entirely clear. Computational modeling suggests that minor cellular heterogeneities could result in widespread network oscillations in rd retina, as has been suggested to occur in other parts of the central nervous system (CNS; Manor et al., 1997). For example, currents flowing through gap junctions would tend to equalize small differences in resting membrane potentials between neighboring AII amacrine cells and/or between ON bipolar and AII amacrine cells (Trenholm et al., 2012). Since changes in membrane potential can lead to the recruitment of the non-linear elements of individual neurons, in such a situation the membrane potential never settles and thus the network oscillates. In such a model oscillations are an emergent property of the gap junction coupled network (Manor et al., 1997; Trenholm et al., 2012; Margolis et al., 2014).

In contrast to the abovementioned network model for oscillations, a recent study has argued that oscillations in the rd retina arise from intrinsic bursting of individual AII amacrine cells, mediated by an interplay between voltage-gated Na^+^ and M-type K^+^ channels (Choi et al., 2014). This study suggests that the primary role for gap junctions is to allow relatively depolarized ON cone bipolar cells to depolarize AII amacrine cells and bring their membrane potential into a state that promotes intrinsic oscillations (Choi et al., 2014). However, while M-type K^+^ channels clearly are important in controlling the kinetics of spontaneous oscillations, the model presented by Choi et al. (2014) is not completely supported by their data: (1) In the rd retina, in the presence of gap junction blockers, AII amacrine cells do not appear to sustain a regular ~10 Hz oscillation when depolarized (compare Figures 6A,B in Choi et al., 2014); (2) Oscillations in AII amacrine cells persist when M-type K^+^ channels are blocked [even in the added presence of voltage-gated Ca^2+^ channel blockers (see Figure 1A; adapted from Figure 9C in Choi et al., 2014)]; (3) Pharmacologically activating M-type K^+^ channels does not block membrane oscillations in ganglion cells in rd retinae (see Figure 10A in Choi et al., 2014). Nonetheless, it should be noted that a limitation of these studies is that they rely on pharmacology to demonstrate the importance of gap junctions in driving network oscillations by indiscriminately blocking gap junctions between AII amacrine cells and other AII amacrine cells, as well as between AII amacrine cells and ON cone bipolar cells. In addition, gap junction blockers can have non-specific effects. For instance, it appears that the widely used gap junction blocker meclofenamic acid can directly activate M-type K^+^ channels (Peretz et al., 2005). Future studies using cell-specific knock out of gap junctions (either exclusively from AII amacrine or ON cone bipolar cells) will help to elucidate precisely how gap junctions in the AII amacrine/ON cone bipolar cell network promote oscillations.

Finally, if TTX-sensitive Na^+^ channels are present in AII amacrine cells in wild type retinae, and AII amacrine cells are extensively coupled to each other as well as to ON cone bipolar cells in wild type retinae, why do these components only contribute to the generation of network wide oscillations in rd retinae? Critical insights into the mechanism underlying oscillations came from pharmacological experiments in wild type retinae showing that hyperpolarizing the AII amacrine/ON cone bipolar cell network by pharmacologically blocking photoreceptor output led to the emergence of ~10 Hz oscillations in wild type AII amacrine/ON cone bipolar cells (Figure 1B; Trenholm et al., 2012). These pharmacologically induced oscillations exhibited the same pharmacological properties as oscillations in the rd retina (Trenholm et al., 2012), suggesting a common underlying mechanism. Similar results have also been found when wild type retinae were bleached with light (Menzler et al., 2014). These finding suggests that no overt changes in the biophysical properties of the AII amacrine/ON cone bipolar cell network are required upon rd to drive oscillations. Instead, it appears that a network hyperpolarization—resulting from the loss of photoreceptor input to bipolar cells—sets the AII amacrine/ON cone bipolar cell network into an oscillatory state. Indeed, direct measurements of the resting potential of AII amacrine cells in the rd retina have revealed that they are more hyperpolarized compared to their wild type counterparts (Choi et al., 2014). Membrane hyperpolarization of the AII amacrine/ON cone bipolar cell network may serve to increase the pool of available Na^+^ channels by bringing them out of an inactivated state, and thereby induce oscillations (Trenholm et al., 2012). Thus, dramatic changes in ganglion cell spike activity during rd appear to arise from relatively moderate changes in the steady-state membrane potential of presynaptic neurons.

## Summary

**Figure 2:**
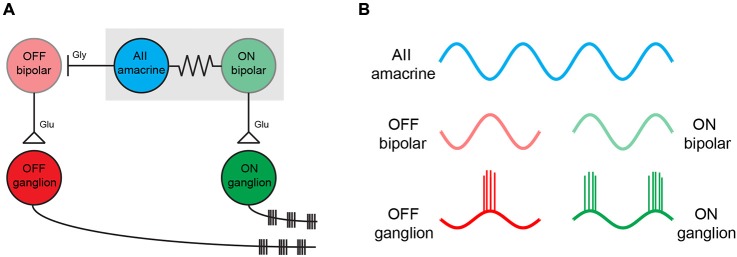
**The simplified circuit diagram for spontaneous oscillations in the rd retina. (A)** A simplified circuit showing the major neuronal cell types that play a role in generating oscillations in the rd retina (adapted from Borowska et al., 2011; Margolis et al., 2014). The oscillation is an intrinsic property of the electrically coupled network of AII amacrine cells and ON cone bipolar cells (indicated with the gray box). AII amacrine cells inhibit OFF bipolar cells with glycine (labeled as gly). ON and OFF bipolar cells activate ON and OFF ganglion cells, respectively, via glutamate release (labeled as glu). Neighboring ON and OFF ganglion cells oscillate out of phase with one another **(B)**. Oscillation in the AII amacrine/ON cone bipolar cell network interact with multiple types of amacrine cells and result in altered dynamics in the ~30 types of ganglion cell microcircuits (not shown).

Taken together, these experiments reveal several key features of the retina following photoreceptor degeneration and these are outlined in Figure 2. First, spontaneous activity increases after the loss of photoreceptor input and leads to rhythmic activity in retinal ganglion cells. The primary source of this spontaneous noise lies in the electrically coupled network of AII amacrine and ON cone bipolar cells. This coupled network appears to become spontaneously active partly due to a small hyperpolarization that occurs upon loss of photoreceptor input during rd. Second, blocking gap junctions inhibits oscillations, meaning that oscillations arise from cross-junctional interactions of conductances between different neurons in the AII amacrine/ON cone bipolar cell network. Third, voltage-gated Na^+^ channels are required for driving the oscillation. Fourth, bipolar cells appear to play a key role in setting the membrane potential of the coupled network into a state in which it can oscillate, and active membrane properties of bipolar cells, including I_h_, regulate the amplitude and frequency of oscillations. Finally, no major rewiring of the retina appears to be required for driving oscillations, as pharmacologically blocking photoreceptor output or photo-bleaching the wild type retina generates similar pacemaker activity. Future work targeting the different components that modulate this spontaneous activity may allow for this spontaneous noise to be dampened in order to heighten the effectiveness of vision restoration strategies. Indeed, such work has already commenced (Toychiev et al., 2013). Additionally, performing similar experiments *in vivo*, such as injecting pharmacological agents into the eye while monitoring spontaneous activity in higher visual centers, will be important for outlining which strategies for mitigating spontaneous retinal activity have the best chance of translating from the lab to the clinic.

## Conflict of Interest Statement

The authors declare that the research was conducted in the absence of any commercial or financial relationships that could be construed as a potential conflict of interest.
